# Uncovering the link between the *SpnIII* restriction modification system and LuxS in *Streptococcus pneumoniae* meningitis isolates

**DOI:** 10.3389/fcimb.2023.1177857

**Published:** 2023-05-01

**Authors:** Hannah N. Agnew, John M. Atack, Ann R.D. Fernando, Sophie N. Waters, Mark van der Linden, Erin Smith, Andrew D. Abell, Erin B. Brazel, James C. Paton, Claudia Trappetti

**Affiliations:** ^1^Research Centre for Infectious Diseases, Department of Molecular and Biomedical Science, University of Adelaide, Adelaide, SA, Australia; ^2^Institute for Glycomics, Griffith University, Gold Coast, QLD, Australia; ^3^School of Environment and Science, Griffith University, Gold Coast, QLD, Australia; ^4^German National Reference Center for Streptococci, University Hospital Rheinisch-Westfälische Technische Hochschule (RWTH) Aachen, Aachen, Germany; ^5^School of Physical Sciences, Faculty of Sciences, Engineering and Technology, University of Adelaide, Adelaide, SA, Australia

**Keywords:** *Streptococcus pneumoniae* (pneumococcus), virulence, clinical isolates bacterial, Quorum sensing, biofilm

## Abstract

*Streptococcus pneumoniae* is capable of randomly switching their genomic DNA methylation pattern between six distinct bacterial subpopulations (A-F) via recombination of a type 1 restriction-modification locus, *spnIII*. These pneumococcal subpopulations exhibit phenotypic changes which favor carriage or invasive disease. In particular, the *spnIIIB* allele has been associated with increased nasopharyngeal carriage and the downregulation of the *luxS* gene. The LuxS/AI-2 QS system represent a universal language for bacteria and has been linked to virulence and biofilm formation in *S. pneumoniae*. In this work, we have explored the link between *spnIII* alleles, the *luxS* gene and virulence in two clinical pneumococcal isolates from the blood and cerebrospinal fluid (CSF) of one pediatric meningitis patient. The blood and CSF strains showed different virulence profiles in mice. Analysis of the *spnIII* system of these strains recovered from the murine nasopharynx showed that the system switched to different alleles commensurate with the initial source of the isolate. Of note, the blood strain showed high expression of *spnIIIB* allele, previously linked with less LuxS protein production. Importantly, strains with deleted *luxS* displayed different phenotypic profiles compared to the wildtype, but similar to the strains recovered from the nasopharynx of infected mice. This study used clinically relevant *S. pneumoniae* strains to demonstrate that the regulatory network between *luxS* and the type 1 restriction-modification system play a key role in infections and may support different adaptation to specific host niches.

## 1 Introduction

*Streptococcus pneumoniae* (the pneumococcus) is a Gram-positive bacterium that asymptomatically colonizes the nasopharynx in up to 95% of infants and 25% of adults (Trimble et al., 2020). However, it can migrate to other niches in the body and cause localized diseases, such as otitis media and sinusitis, or it can disseminate to sterile sites to cause invasive pneumococcal diseases (IPD) such as pneumonia, bacteremia and meningitis. The broad range of both localized and invasive diseases it causes, results in more than 190 million infections with 1.2 million deaths world-wide each year, making it one of the most pathogenic bacterial species (Lavelle and Ward, 2022).

The mechanism by which *S. pneumoniae* transit from colonizer to pathogen is not well understood. However, pneumococci are capable of randomly switching their genomic DNA methylation pattern between six distinct bacterial subpopulations (A-F) via recombination of a type 1 restriction-modification locus, *spnIII*, with any given culture comprising a mixture of genotypes. These pneumococcal subpopulations exhibit phenotypic changes which favor carriage or invasive disease. Of particular interest, the *spnIIIA* allele was found to be associated with higher quantities of opaque phase pneumococcal colonies and had a more invasive phenotype. In contrast, the *spnIIIB* allele has been associated with increased nasopharyngeal carriage and the downregulation of the *luxS* gene (Manso et al., 2014).

LuxS is an essential component of the “universal” Quorum sensing (QS) LuxS/Autoinducer-2 (AI-2) system present in both Gram-positive and Gram-negative bacteria (Miller and Bassler, 2001; Von Bodman et al., 2008). The metabolic enzyme LuxS (an S-ribosylhomocysteine lyase) synthesizes AI-2 as a by-product of the conversion of S-ribosyl-homocysteine to homocysteine, an essential reaction of the activated methyl cycle. There are numerous QS systems, each allowing bacteria to detect stimuli, through autoinducers, and respond by inducing changes in gene expression of target cells (De Kievit and Iglewski, 2000). Although, the response to the LuxS/AI-2 system has been well characterized in Gram-negative bacteria (Vendeville et al., 2005), it is not well understood in Gram-positive bacteria, and in particular *S. pneumoniae*. Importantly in pneumococci, the LuxS/AI-2 QS system has been linked to virulence and biofilm formation (Stroeher et al., 2003; Trappetti et al., 2011a).

Biofilms can be defined as highly complex and dynamic surface associated structures, which provide *S. pneumoniae* a means of evading the host immune response and antimicrobial treatment, making biofilms indispensable for the persistence of pneumococcal disease (Costerton et al., 1999; Kolenbrander, 2000). In the laboratory strain D39, addition of extracellular AI-2 induced biofilm formation, with a deletion of the *luxS* gene resulting in decreased biofilm formation (Trappetti et al., 2011b) and decreased virulence in a murine model (Stroeher et al., 2003). The effect of *luxS* expression on biofilm formation has also been assessed in clinical isolates (Trappetti et al., 2013; Tikhomirova et al., 2022). We have previously found that serotype 3 pneumococcal isolates from the blood of 12 distinct patients had higher levels of *luxS* expression compared with isolates from the ears of 13 distinct patients, indicating that the role of *luxS* in biofilm formation may differ depending on the site of isolation (Trappetti et al., 2013). Recently we have investigated the role of LuxS in the middle ear isolate 947 serotype 14 sequence type (ST) 15 (Tikhomirova et al., 2022). We found that while biofilm formation was similar between the wildtype 947 strain and the *luxS* deletion mutant, the *luxS* mutant appeared more virulent than the parent strain in an intranasal murine infection model. These findings suggest that LuxS/AI-2 QS system in the middle ear isolate may have an opposite effect to that of the invasive strain D39 (Stroeher et al., 2003; Tikhomirova et al., 2022).

In the current study, we have characterized *luxS* in two *S. pneumoniae* clinical isolates obtained at the same time from the blood and cerebral spinal fluid (serotype 15C, ST 8711) of a single pediatric meningitis patient. Previously we have shown that these clinical isolates display different virulence phenotypes in a murine infection model (Agnew et al., 2022). Here, we aimed to investigate the role of *luxS* in these two clinical isolates.

## 2 Materials and methods

### 2.1 Bacterial strains and growth conditions

The wildtype *S. pneumoniae* strains used in this study (**Table 1**) were provided to us by the German National Reference Center for Streptococci (Aachen) (Agnew et al., 2022). Pneumococci serotype 15C blood and CSF isolates (designated 60B and 60CSF, respectively) were isolated in 2015 from a child, aged 2, admitted to hospital with meningitis. The cells were routinely grown on Columbia agar supplemented with 5% (vol/vol) horse blood (BA), with or without gentamicin (5 µg/mL) or spectinomycin (200 μg/mL), at 37°C in 5% CO_2_ overnight. Growth assays were carried out using pneumococci grown in a chemically defined media (CDM) which is composed of SILAC RPMI 1640 Flex Media supplemented with amino acids, vitamins, choline chloride and catalase (10 U/mL) as described previously (Agnew et al., 2022), and either 0.5% (w/v) glucose or 0.5% (w/v) galactose.

**Table 1:** *S. pneumoniae* strains and oligonucleotide primers used in this study.

Strain or primer	Description or sequence (5’➔3’)	Source or reference
Strains		
60B	Clinical isolate from blood (serotype 15C, sequence type 8711) (SN69534)	Agnew et al., 2022
60CSF	Clinical isolate from cerebral spinal fluid (serotype 15C, sequence type 8711) (SN69531)	Agnew et al., 2022
60BΔluxS	*luxS* deletion-replacement mutant (spec^r^)	This study
60CSFΔluxS	*luxS* deletion-replacement mutant (spec^r^)	This study
Primers		
Oligo name	Sequence (5’ ➔ 3’)	Reference
*luxS for*	TGGACCAGCCCTAGCCTTTGAA	Trappetti et al., 2017
*luxS Rev*	CACACTTGACTAAGGAAGAC	Trappetti et al., 2017
*luxS spec for*	AAATAACAGATTGAAGAAGGTATAATCTCACACCACCGTACGTA	Trappetti et al., 2017
*luxS spec Rev*	TATGTATTCATATATATCCTCCTCGTTGCTCCTGAGACAGA	Trappetti et al., 2017
*luxS-RT-F*	CCCTATGTTCGCTTGATTGGGG	Trappetti et al., 2011b
*luxS-RT-R*	AGTCAATCATGCCGTCAATGCG	Trappetti et al., 2011b
*gyr-RT-F*	ACTGGTATCGCGGTTGGGAT	Mclean et al., 2020
*gyr-RT-R*	ACCTGATTTCCCCATGCAA	Mclean et al., 2020

### 2.2 Construction of mutant strains

The *luxS* gene was deleted from *S. pneumoniae* 60B and 60CSF strains and replaced with a spectinomycin resistance cassette by transformation. A linear DNA fragment containing the resistance cassette was constructed by overlap extension PCR (Iannelli and Pozzi, 2004), using primers listed in **Table 1**.

### 2.3 Growth assays

The strains were grown in flat-bottom 96-well microtiter plates (Costar) with a final volume of 200 µL as previously described (Minhas et al., 2019). Bacterial strains were inoculated at a starting optical density at 600 nm (OD_600_) of 0.05 in CDM supplemented with either 0.5% (w/v) glucose or 0.5% (w/v) galactose and AI-2 (refer to Supplementary data for synthesis of AI-2) at concentrations of 4 µM, 10 µM, 100 µM and 200 µM, then incubated at 37°C with 5% CO_2_. The OD_600_ was measured every 30 min for a total of 24 h in a SpectroSTAR Omega spectrophotometer (BMG Labtech). Assays were conducted in triplicate with at least two repeated independent experiments. Statistically significant differences in both final OD_600_ and mid-exponential phase OD_600_ between strains was determined using two-tailed Student’s *t* test; *P* < 0.05 were deemed statistically significant.

### 2.4 RNA extraction and qRT-PCR

Strains were grown overnight on BA plates at 37°C with 5% CO_2_. Cells were harvested, washed and resuspended in 1 mL of CDM to a final OD_600_ of 0.2. Bacterial suspensions were incubated at 37°C with 5% CO_2._ RNA extractions were carried our using a Qiagen RNeasy Minikit as per the manufacturer’s instructions. Differences in gene expression levels were determined using one-step relative real-time qRT-PCR in a Roche LC480 real-time cycler, as described previously (Minhas et al., 2019). Primers used for *luxS* and *gyrA* (internal control) are listed in **Table 1** and were used at a final concentration of 200 nM per reaction. Amplification data were analyzed using the comparative critical threshold (2^-ΔΔCT^) method (Livak and Schmittgen, 2001). Assays were performed in triplicate and statistical analyses were performed using two-tailed Student’s *t* test; *P* values < 0.05 were deemed as statistically significant.

### 2.5 Phenotypic microarrays

Carbon phenotype microarray (PM) analysis using the PM microplate PM1 (Biolog Inc.) was performed on the strains to test for catabolism of 95 different carbon sources as previously described (Minhas et al., 2019). Each well in the microplate contained a different carbon source. The cells were inoculated to a final OD_590_ of 0.06 in the buffer provided, in accordance with the manufacturer’s instructions. This suspension was added in 100 µL aliquots to the wells, and the plate was incubated at 37°C, 5% CO_2_. The OD_590_ was measured every 15 min for 24 h. Catabolic activity was measured through colorimetric analysis, in which a colorless tetrazolium dye was reduced by NADH produced during catabolism. The level of metabolism for each carbon source was arbitrarily determined by comparison with the zero carbon source blank.

### 2.6 Biofilm assays

Biofilm formation was measured in real-time with the real-time cell analyzer (RTCA) xCELLigence (Agilent Technologies Inc.) instrument as previously described (Agnew et al., 2022; Tikhomirova et al., 2022). This instrument detects variations in the impedance signal (expressed as the arbitrary cell index, CI) as bacterial cells attach and form biofilms on the gold-microelectrodes present at the bottom of the E-plates (Agilent Technologies Inc.). Bacterial strains were grown overnight on BA plates at 37°C with 5% CO_2_. The cells were harvested and resuspended in 200 µL of CDM + 0.5% (w/v) glucose or 0.5% (w/v) galactose to a final OD_600_ of 0.2. To the wells of the E-plate, 150 µL CDM + 0.5% (w/v) glucose or 0.5% (w/v) galactose ± 10µM AI-2 was added, before being placed in the cradle of the RTCA-DP system, within a 37°C incubator with 5% CO_2_ supplementation. An initial baseline impedance reading was taken before the E-plates were removed and 50 µL of bacterial suspension added to the wells for a 1 in 4 dilution, reducing the starting OD_600_ to 0.05. An additional 50 µL of CDM + 0.5% (w/v) glucose or 0.5% (w/v) galactose ± 10 µM AI-2 was added to the control wells. The E-plates were locked into the cradles of the RTCA-DP platform within the incubator and the impedance signal (CI) was recorded every 15 min for 24 h to monitor biofilm formation. Assays were conducted in duplicate with at least two repeated independent experiments. Statistical analysis was carried out using a two-tailed Student’s *t* test; *P* values < 0.05 were deemed statistically significant.

### 2.7 Adherence and invasion assays

Adherence and invasion assays were carried out using the Detroit 562 human pharyngeal cell line as previously described (Trappetti et al., 2011a; Amin et al., 2015). Detroit 562 human pharyngeal cells were grown in Dulbecco’s modified Eagle’s medium (DMEM) supplemented with 10% fetal calf serum (FCS), 100 U/mL penicillin and 100 µg/mL streptomycin at 37°C in 5% CO_2_. Wells of 24-well tissue culture trays were seeded with Detroit cells in DMEM with 10% FCS as described previously (Trappetti et al., 2011a) and left to grow overnight. Cells were inoculated with 500 µL of each bacterial suspension grown overnight on BA plates and resuspended in CDM ± 10µM AI-2 at a final OD_600_ of 0.2. The same volume of each bacterial strain was added to empty wells as a control. Adherence assays were conducted after incubation of the bacteria with the Detroit cells for 2 h at 37°C. The wells were washed 3 times with PBS, the cells were detached from the plate by treatment with 100 µL 0.25% trypsin-0.02% EDTA and 400 µL of 0.1% Triton X-100 (Sigma), and samples were plated on BA plates to determine the number of adherent bacteria. Invasion assays were carried out essentially as described above. After the post-adherence washing step, cultures were incubated for 1 h in fresh media supplemented with 200 µg/mL gentamicin and 10 µg/mL penicillin to kill extracellular bacteria. Monolayers were again washed, lysed, serially diluted, and plated on BA, as described above. The assays were conducted in triplicate with at least two repeated independent experiments. Statistical analysis was carried out using a two-tailed Student’s *t*-test; *P* values < 0.05 were considered statistically significant. Data collected are presented as mean adherent or invasive bacteria ± standard error mean (SEM) in CFU/mL. The controls were used to monitor bacterial growth during the 2 h incubation period, ensuring strains grew at similar rates.

### 2.8 Murine infection model

Animal experiments were approved by the University of Adelaide Animal Ethics Committee (approval number S-2022-029). Female outbred 4- to 6-week-old CD-1 (Swiss) mice were anaesthetized by intraperitoneal injection of ketamine (8 mg/mL) and xylazine (0.8 mg/mL), and were challenged intranasally with 50 µL of bacterial suspension containing 1 x 10^8^ CFU in serum broth (SB) as previously described (Minhas et al., 2019). The challenge dose was retrospectively confirmed by serial dilution and plating on BA. At 24 h, groups of 8 mice were euthanized by CO_2_ asphyxiation before the blood, lungs, nasal tissue, ears, and brain were collected. Tissue samples were homogenized in 1 mL PBS, serially diluted and plated on BA plates containing 5 µg/mL gentamicin to enumerate pneumococci as previously described (Trappetti et al., 2011a). Statistical analyses of log-transformed CFU data were performed using two-tailed Student’s *t* test; *P* values < 0.05 were deemed statistically significant.

### 2.9 *spnIII* allele quantification

Female outbred 4- to 6-week-old CD-1 (Swiss) mice were anaesthetized and intranasally challenged as described above. The nasal tissue was harvested 24 h post infection, processed and plated as above. DNA extraction was performed on the *luxS* mutants, WT original challenge inoculum and colonies grown overnight on BA plates supplemented with 5 µg/mL gentamicin, using a Qiagen DNeasy Blood & Tissue kit as per the manufacturer’s instructions. The variant *spnIII* alleles were quantified as previously described (Manso et al., 2014). Statistical analyses were performed using two-way analysis of variance and a Tukey post-comparison test; *P* values < 0.05 were deemed statistically significant.

### 2.10 Isolation of bacteria from nasopharynx of infected mice

Female outbred 4- to 6-week-old CD-1 (Swiss) mice were anaesthetized and intranasally challenged as described above. The nasal tissue was harvested 24 h post infection, processed and plated as above. Bacterial colonies from each mouse (n = 5 for 60CSF, n = 6 for 60B) were resuspended in serum broth and stored at -80°C. Biolog assays were performed as above on bacteria from a single mouse, representative for each strain.

## 3 Results

### 3.1 Blood and CSF strain have different proportions of *spnIII* alleles

**Figure 1:**
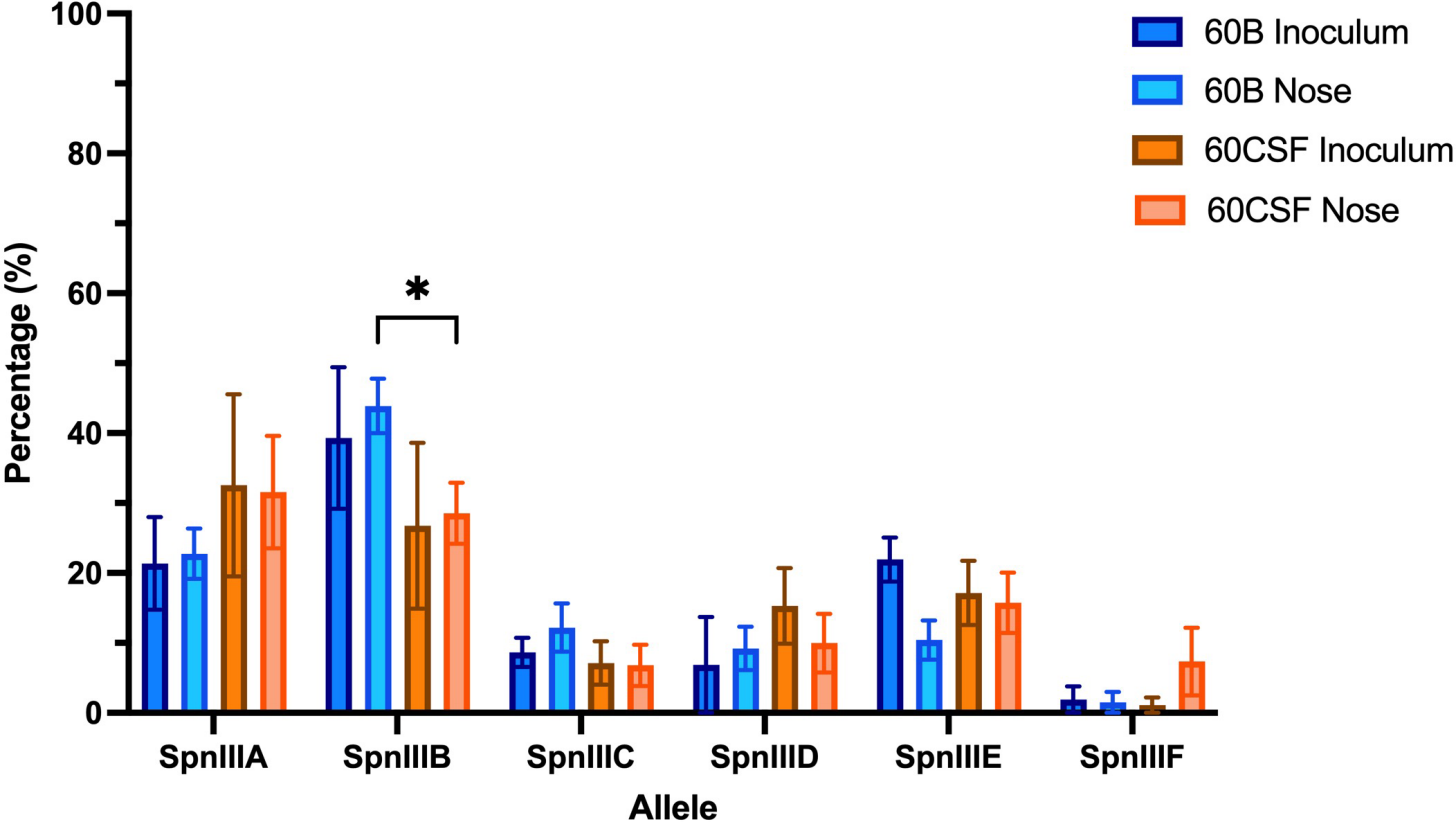
*spnIII* allele frequencies of 60B and 60CSF recovered post intranasal murine infection. Mice were intranasally challenged with 10^8^ CFU *S. pneumoniae* serotype 15C ST8711 blood isolate (60B) or CSF isolate (60CSF). At 24 h, mice from each group were humanely euthanized and pneumococci in the nasal tissue were harvested. *spnIII* allele quantification was performed on DNA extracted from the original inoculum and colonies grown from nasopharynx samples. Allele percentages for original inoculum and bacteria recovered from noses are represented. Statistical analysis was performed using two-way analysis of variance and a Tukey post-comparison test; *P < 0.05.

Previously, we have shown that while the CSF clinical *S. pneumoniae* isolate (60CSF) is found in the ears of infected mice 24 h post intranasal infection, the blood isolate (60B) was unable to survive in this niche (Agnew et al., 2022). To determine whether the observed different virulence phenotypes of 60B and 60CSF in mice could be mediated by rearrangements in the *spnIII* Type I restriction-modification (RM) system, the *spnIII* allele distribution was assessed from pneumococcal DNA extracted from the nasopharynx of intranasally infected animals. Mice were intranasally challenged with 10^8^ colony forming units (CFU) of each strain and the bacteria were taken from the nose 24 h post-infection. The DNA was isolated and the *spnIII* alleles were quantified for the inoculum and recovered bacteria for both strains. The 60B inoculum used to challenge the mice had predominantly *spnIIIB* (21% *spnIIIA*, 39% *spnIIIB*, 9% *spnIIIC*, 7% *spnIIID*, 22% *spnIIIE* and 2% *spnIIIF*), while the 60CSF inoculum was skewed towards *spnIIIA* (33% *spnIIIA*, 27% *spnIIIB*, 7% *spnIIIC*, 15% *spnIIID*, 17% *spnIIIE* and 1% *spnIIIF*) (**Figure 1**). The bacteria recovered from the nasopharynx of both groups of mice, showed no change in the *spnIII* allele distribution. The *spnIIIB* allele remained the predominant allele in all samples from the nasopharynx of mice challenged with 60B (23% *spnIIIA*, 44% *spnIIIB*, 12% *spnIIIC*, 9% *spnIIID*, 10% *spnIIIE* and 2% *spnIIIF*), while mice challenged with 60CSF, showed a similar proportion of *spnIIIA* and *spnIIIB* (32% *spnIIIA*, 28% *spnIIIB*, 7% *spnIIIC*, 10% *spnIIID*, 16% *spnIIIE* and 7% *spnIIIF*). Interestingly, when comparing the bacteria recovered from the noses of infected mice, 60B had a significantly higher proportion of the *spnIIIB* allele compared to 60CSF (**Figure 1**).

### 3.2 Downregulation of the *luxS* gene in 60B strain

**Figure 2:**
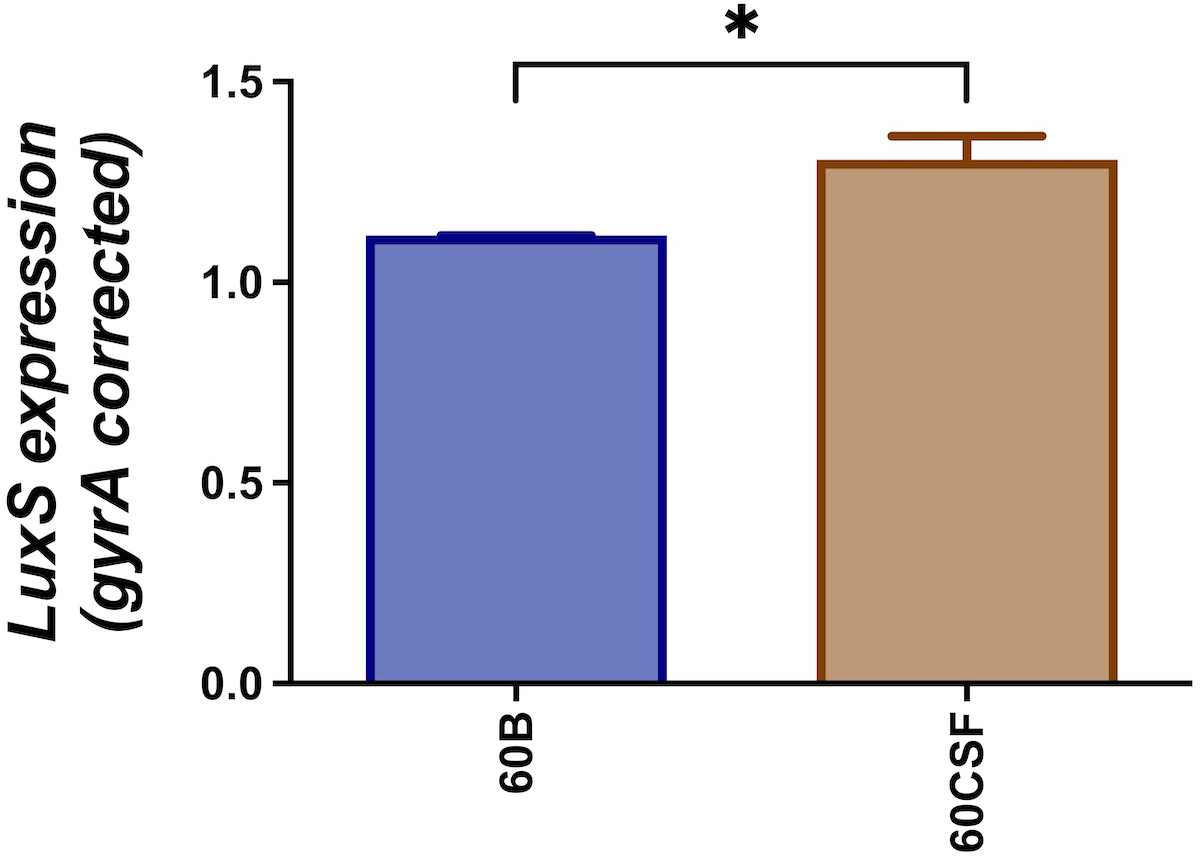
Expression of *luxS* by 60B and 60CSF isolates. Strains were resuspended in CDM at OD_600_ 0.2 and incubated at 37°C with 5% CO_2_ for 30 min. RNA was extracted and *luxS* mRNA levels were analyzed by qRT-PCR using *gyrA* rRNA as an internal control (see Materials and Methods). Data are mean OD_600_ ± standard error mean (SEM) from three biological replicates. Significance of differences in gene expression between isolates was determined using two-tailed Student’s t test; *P < 0.05.

We have previously reported that cell-cell signaling via the autoinducer 2 (AI-2)/LuxS quorum-sensing system is linked to the *spnIIIB* variant in *S. pneumoniae*. In particular, the *luxS* gene was found to be downregulated in SpnD39IIIB locked strain compared to the other SpnD39III locked strains (Manso et al., 2014). As reported above, the 60B strain has higher proportion of *spnIIIB* allele, while 60CSF has comparable level of *spnIIIA* and *spnIIIB* allele (**Figure 1**). Thus, we analyzed *luxS* gene expression in the 60B and 60CSF strains. Importantly, we found downregulation of the *luxS* gene in the 60B strain in which the *spnIIIB* allele is the predominant allele (**Figure 2**).

### 3.3 *luxS* mutants displayed similar metabolic profile of strains recovered from the nasopharynx of infected mice

LuxS regulates key aspects of carbohydrate metabolism in *S. pneumoniae* and the capacity to utilize different carbon sources is crucial for nasopharyngeal colonization (Minhas et al., 2019; Mclean et al., 2020; Agnew et al., 2022). Thus, we employed a phenotypic microarray to compare the capacity of strains recovered from the nasopharynx of mice infected with either 60B or 60CSF to metabolize 95 different carbohydrates (see Materials and Methods). Interestingly, strains recovered from the nose of mice infected with either 60B or 60CSF showed a distinct metabolic profile to that of the inoculum strains. Importantly, the nasopharyngeal strains were now able to metabolize L-fucose, but unable to metabolize D-psicose (**Supplementary Table 1**).

We then created a *luxS* deletion mutant in both 60B and 60CSF. The metabolic profiles of these mutants were then assessed with the phenotypic microarray. Surprisingly, we found that these strains paralleled the metabolic profiles of the strains recovered from mice and not that of the original inoculating strains (**Supplementary Table 1**). These results indicate that the strains may undergo a change in the nose of mice that results in an increase of the *spnIIIB* allele to aid their colonization and resulting in reduced *luxS* expression, consequently leading to a similar metabolic profile to that of the *luxS* mutants. Thus, there appears to be an unknown link between LuxS and the *spnIIIB* allele, which merits further investigation.

### 3.4 The *luxS* mutants display a dramatic shift in proportion of *spnIII* alleles

**Figure 3:**
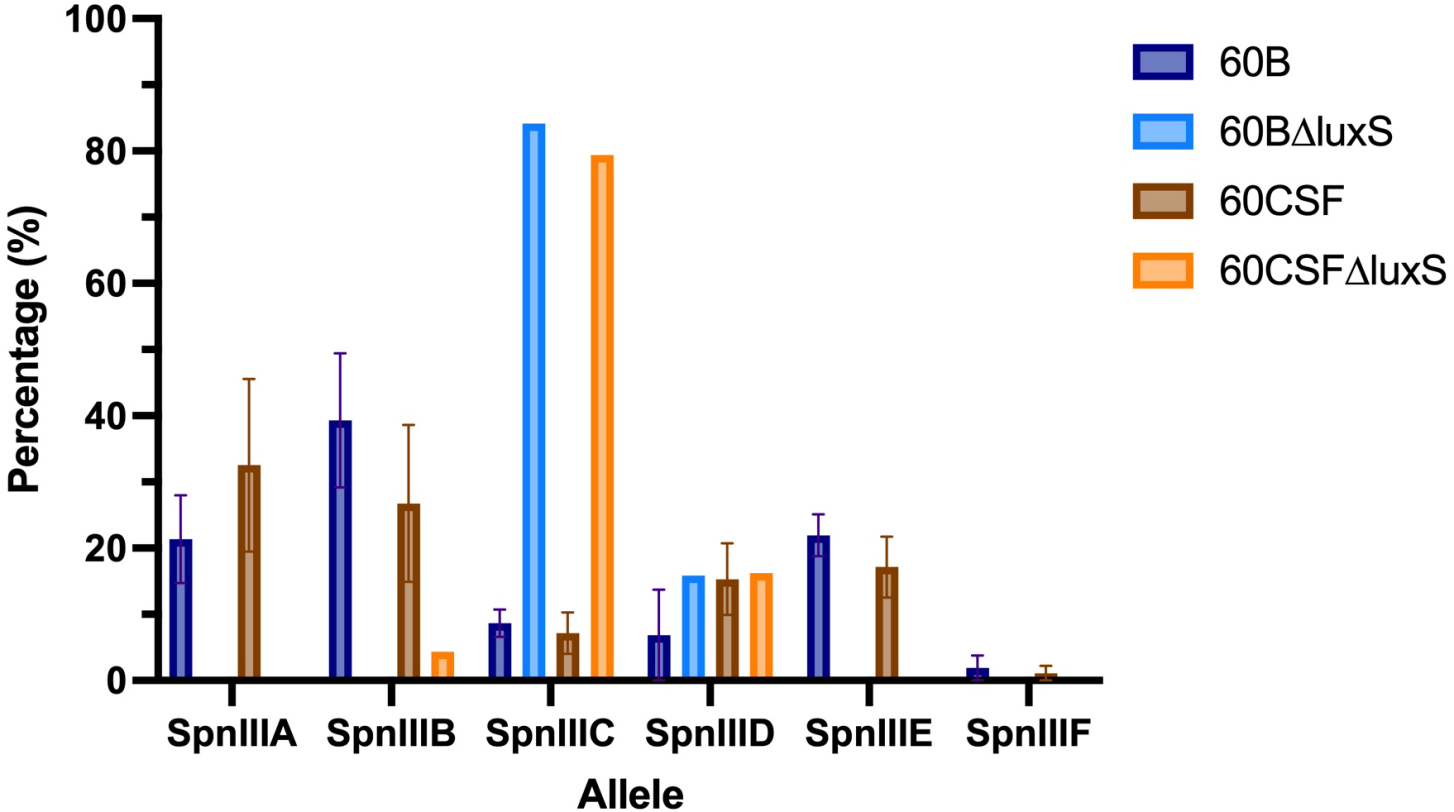
*spnIII* allele frequencies of 60B, 60CSF and *luxS* mutants. *spnIII* allele quantification was performed on DNA extracted from 60B, 60CSF, 60B∆luxS and 60CSF∆luxS colonies grown on BA plates overnight at 37°C with 5% CO_2_. Allele percentages are displayed for WT original inoculum (**Figure 1**) and *luxS* mutants from frozen stock.

Based on the results obtained in Section 3.3, we investigated the *spnIII* allele profiles of the *luxS* deletion mutants in 60B and 60CSF. As shown in **Figure 3**, it was found that 60BΔluxS possessed only *spnIIIC* and *spnIIID* (0% *spnIIIA*, 0% *spnIIIB*, 84.15% *spnIIIC*, 15.85% *spnIIID*, 0% *spnIIIE* and 0% *spnIIIF*), similar to 60CSFΔluxS (0% *spnIIIA*, 4.36% *spnIIIB*, 79.39% *spnIIIC*, 16.25% *spnIIID*, 0% *spnIIIE* and 0% *spnIIIF*). Compared to the WT 60B and 60CSF inoculums, both *luxS* mutants had no *spnIIIA* present and *spnIIIB* was only present in 60CSF∆luxS at a very low proportion. Interestingly, we found both mutants were now predominantly expressing *spnIIIC*. Similar results were seen when comparing the *luxS* deletion mutants with the WT strains recovered from the nose of infected mice, in which the *luxS* mutants had more *spnIIIC*, little to no *spnIIIB* and no *spnIIIA* (**Supplementary Figure 1**). These results support the link between LuxS and the *spnIII* locus, with a complete absence of LuxS resulting in a shift towards high proportions of *spnIIIC* and a severe decrease in *spnIIIA* and *spnIIIB.*

### 3.5 *luxS* has a different impact in the 60B and 60CSF strains

**Figure 4:**
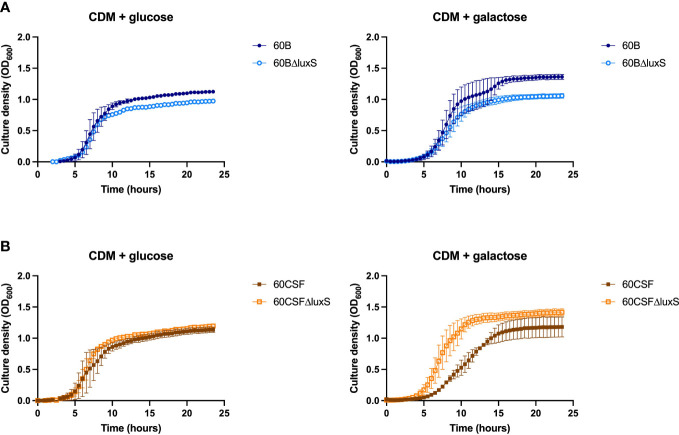
Growth of 60B, 60CSF and *luxS* mutants in CDM + glucose and CDM + galactose. 60B and its *luxS* mutant 60BΔluxS **(A),** and 60CSF and its *luxS* mutant 60CSFΔluxS **(B)** were grown in 200 µL CDM supplemented with 0.5% glucose (CDM + glucose) or 0.5% galactose (CDM + galactose). OD_600_ was measured every 30 min for 24 (h) Data are mean OD_600_ ± standard error mean (SEM) from two independent assays, each performed in triplicate.

*S. pneumoniae* is unable to grow with fucose as the sole carbon source, therefore, none of our established assays could be used for further analysis (Higgins et al., 2014). Similarly, D-Psicose is a low energy hexoketose monosaccharide, which is a C-3 epimer of D-fructose and is rarely found in nature, thus it is unlikely to play a role in pneumococcal virulence (Matsuo et al., 2002). Therefore, to further characterize our *luxS* mutant we used a different approach. LuxS QS boosts the capacity of *S. pneumoniae* to utilize galactose as a carbon source by upregulation of the Leloir pathway, so we then assessed the capacity of the *luxS* mutants to grow in galactose. Glucose was used as a control sugar, as it is the major sugar present in blood (Paixão et al., 2015). In CDM + glucose or galactose, wildtype (WT) strains showed comparable growth (**Supplementary Figure 2**). In **Figure 4A**, in CDM + glucose the 60B *luxS* deletion mutant (60BΔ*luxS*) had a slight reduction in the final cell density (*P* value = 0.0329), while in CDM + galactose the observed reduction is more prominent (*P* value = 0.0257). Interestingly, the 60CSF *luxS* deletion mutant (60CSFΔ*luxS*) showed comparable growth to that of the WT 60CSF in CDM + glucose (**Figure 4B**; *P* value = 0.3057), but an increased generation time compared to 60CSF in CDM + galactose was observed (*P* value = 0.0493). These results show that *luxS* deletion has a different impact in the two closely related strains.

### 3.6 Higher doses of AI-2 negatively impacts growth of 60CSF

**Figure 5:**
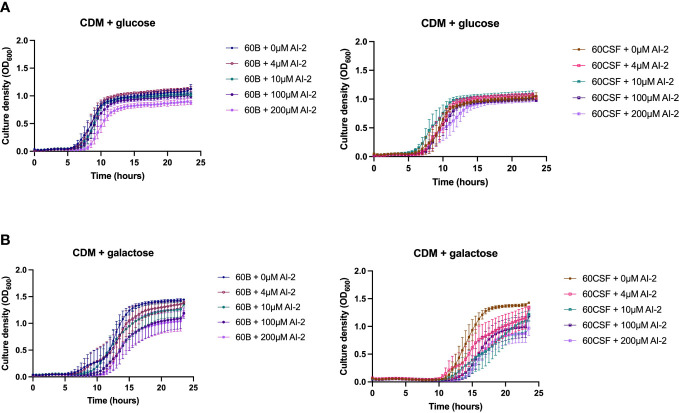
Growth of 60B and 60CSF with AI-2 supplementation at different concentrations. 60B and 60CSF were cultured in CDM + glucose **(A)** or CDM + galactose **(B)** supplemented with 0, 4, 10, 100 or 200 µM. OD_600_ was measured every 30 min for 24 (h) Data are mean OD_600_ ± standard error mean (SEM) from three independent assays, each performed in triplicate.

It has previously been demonstrated that the addition of 10 µM exogenous AI-2 was able to moderately restore the defective growth of a D39 *luxS* deletion strain (Trappetti et al., 2017). However, it was also shown in two serotype 14 blood and ear isolates that the addition of exogenous AI-2 reduces the growth in a dose-dependent manner (Tikhomirova et al., 2022). Therefore, the effects of different concentrations of exogenous AI-2 on the growth of the blood (60B) and CSF (60 CSF) strains was assessed. In CDM + glucose, for both 60B and 60CSF strains the addition of 4 and 10 µM AI-2 had little or no effect on growth, but at the higher concentrations of 100 µM and 200 µM AI-2, reduction in growth was observed (**Figure 5A**). Interestingly, in CDM + galactose the addition of AI-2 caused reduced growth of 60CSF for all concentrations (4, 10, 100 and 200 µM) tested, whilst in 60B the reduced growth was only different when higher concentration of AI-2 (100 and 200 µM) were added to the media (**Figure 5B**).

### 3.7 Addition of exogenous AI-2 enhances growth in 60B *luxS* deletion mutant

**Figure 6:**
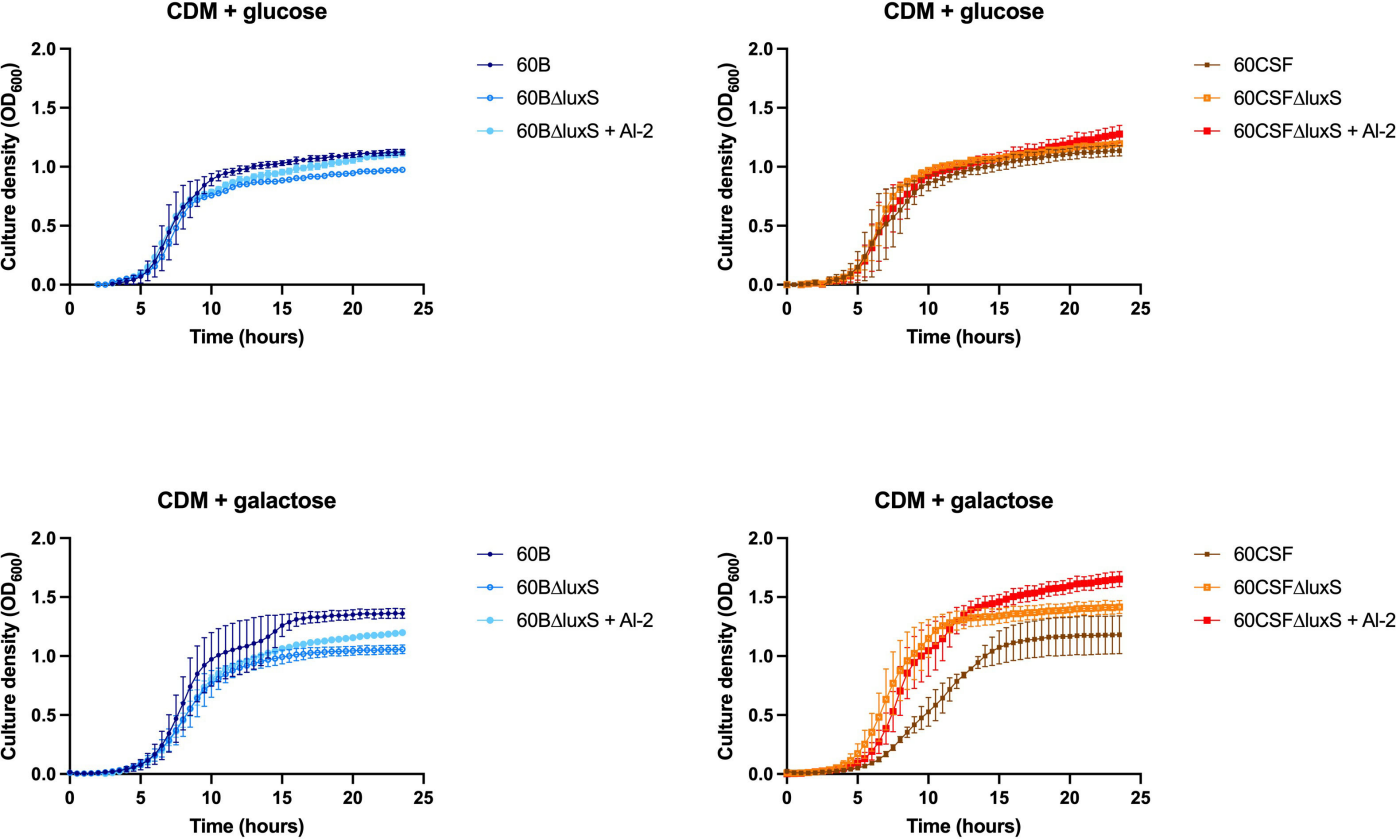
Impact of AI-2 on growth of *luxS* mutants. 60B, 60CSF, 60BΔluxS and 60CSFΔluxS were grown in 200 µL CDM supplemented with 0.5% glucose (CDM + glucose) or 0.5% galactose (CDM + galactose). OD_600_ was measured every 30 min for 24 h. Data are mean OD_600_ ± standard error mean (SEM) from two independent assays, each performed in triplicate.

As the addition of AI-2 caused a growth defect in the WT 60B and 60CSF strains (**Figure 5**), we examined the capacity of exogenous AI-2 to complement the growth defect of *luxS* deletion strain. In **Figure 6**, addition of exogenous AI-2 to 60BΔ*luxS* and 60CSFΔ*luxS* had no effect in CDM + glucose, while it enhanced the growth of the 60BΔ*luxS* mutant in CDM + galactose. These results confirmed a dose-dependent effect previously observed in the D39 strain (Trappetti et al., 2017).

### 3.8 *luxS* plays a key role in the blood and CSF strains’ ability to form a biofilm

**Figure 7:**
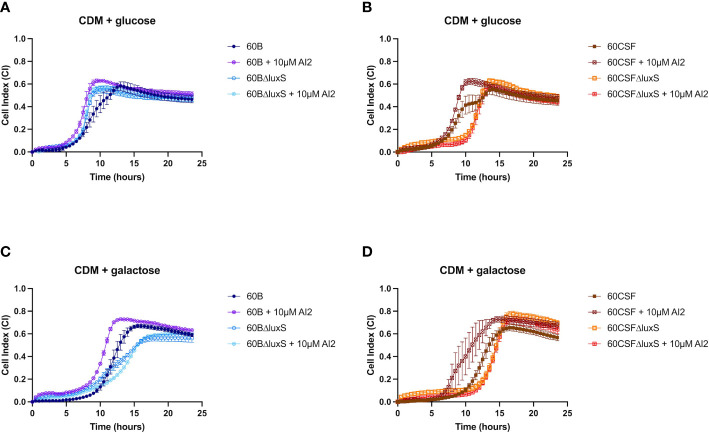
Biofilm formation of 60B, 60CSF, 60BΔluxS and 60CSFΔluxS strains. Bacteria were cultured overnight on BA plates before being diluted to a final OD_600_ of 0.05 in CDM + 0.5% glucose ± 10 µM AI-2 **(A, B)** or CDM + 0.5% galactose ± 10 µM AI-2 **(C, D)**. 200 µL of each bacterial strain in each culture medium was placed into wells of a xCELLigence E-plate, with the plate being placed in the cradles of the RTCA-DP platform and incubated at 37°C with 5% CO_2_. Biofilm formation was determined by measuring cell index (CI) every 15 min over 24 h using real-time cell analysis (RTCA) xCELLigence technology. Data are mean CI ± standard error mean (SEM) from three independent assays, each performed in triplicate.

Previously, *luxS* has been shown to play a key role in the ability of D39 to form a biofilm (Trappetti et al., 2011b). Therefore, we assessed the ability of WT 60B, 60CSF and *luxS* deletion mutants to form biofilm in real-time using CDM + glucose and CDM + galactose, with or without supplementation of 10 µM AI-2. In CDM + glucose and CDM + galactose both 60B and 60CSF showed the same capacity to form a biofilm (**Supplementary Figure 3**). The 60BΔ*luxS* mutants formed a biofilm at a slightly increased rate compared to WT 60B in CDM + glucose (**Figure 7A**). Interestingly, the 60CSFΔ*luxS* mutant had delayed biofilm formation compared to WT in CDM + glucose (**Figure 7B**). Addition of exogenous AI-2 greatly enhanced biofilm formation of WT strains but had no impact on *luxS* mutant strains (**Figures 7A, B**). In CDM + galactose, both *luxS* mutants (60BΔ*luxS* & 60CSFΔ*luxS*) had delayed biofilm formation compared to their WT counterparts (**Figures 7C, D**), with 60CSFΔ*luxS* forming more biofilm than WT 60CSF. Similar to the earlier results in glucose, addition of AI-2 increased the biofilm formation of the WT 60B and 60CSF strains whilst appearing to have no impact in the *luxS* mutants.

*luxS* has been found to be crucial for adherence to epithelial cells (Tikhomirova et al., 2022). Therefore, we investigated the role of LuxS in adherence to, and invasion of, human epithelial cells, using the human nasopharyngeal Detroit 562 cell line in CDM with or without 10 µM AI-2 supplementation. No significant differences in the total number of adherent and invasive cells were observed between the WT strains and the *luxS* deletion mutants in CDM, with or without the addition of AI-2 (**Supplementary Figure 4**). These results are similar to those previously found for a D39 *luxS* deletion mutant in which there was no difference between WT and mutant in adherence to lung (A549) or larynx (HEp-2) derived cell lines (Stroeher et al., 2003).

### 3.9 Deletion of *luxS* in blood and CSF strains affects virulence in a murine model

**Figure 8:**
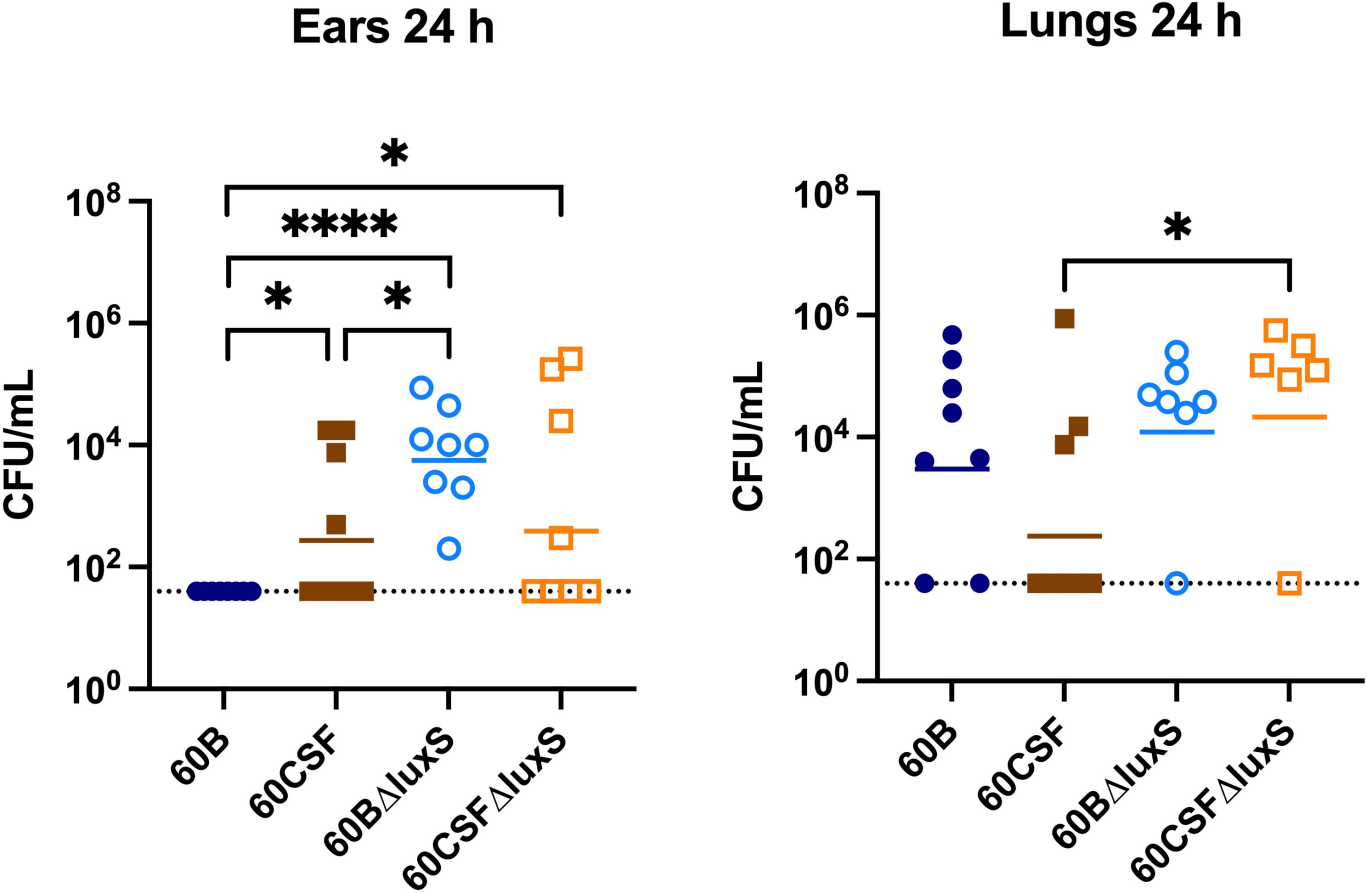
Virulence phenotypes of 60B, 60CSF, 60BΔluxS and 60CSFΔluxS strains. Groups of 8 mice were infected intranasally with 10^8^ CFU of the indicated strain. At 24 h, mice from each group were humanely euthanized and pneumococci in the brain, ear, lungs and nasal tissue were enumerated. Viable bacterial counts are displayed for each mouse in the ears and lungs (brain and nose data in **Supplementary Figure 5**); horizontal bars indicate the geometric mean (GM) CFU for each group; the dotted line indicates the detection threshold. Significance of differences in bacterial load between groups was determined using two-tailed Student’s *t* test; *P < 0.05, ****P < 0.0001.

The ability of *S. pneumoniae* to metabolise specific carbohydrates has previously been linked to pneumococci disease phenotype in a murine model (Minhas et al., 2019; Agnew et al., 2022). As *luxS* may play a role in carbohydrate metabolism in pneumococci, their virulence phenotypes were assessed in a murine model. It has previously been shown that *luxS* plays a role in the pathogenesis of *S. pneumoniae* D39 and ear isolate 947 (Stroeher et al., 2003; Tikhomirova et al., 2022). Specifically, in the 947 strain, deletion of *luxS* resulted in a significantly higher bacterial burden in the ears of infected mice (Tikhomirova et al., 2022). An earlier study of the blood and CSF strains has shown that they behave differently in a murine model, whereby 60B was unable to survive in the ear (Agnew et al., 2022). Here, we extended these studies by exploring the 60B and 60CSF *luxS* deletion strains in our intranasal murine infection model. CD-1 Swiss mice were intranasally challenged with 10^8^ CFU of each strain (60B, 60CSF, 60BΔ*luxS* & 60CSFΔ*luxS*) and the bacterial burden in the blood, brain, ears, lungs, and nose was assessed at 24 h post challenge. At 24 h post infection, there were no significant differences observed in the bacterial burdens in the brain and nasopharynx between mice infected with WT or *luxS* deletion strains (**Supplementary Figure 5**). Consistent with previous work (Agnew et al., 2022), no bacteria were detected in the ears of mice challenged with 60B, whilst bacteria were present in the ears of 50% of the mice infected with 60CSF (**Figure 8**; *P* < 0.05). Strikingly, the bacterial burden in the ears of mice challenged with 60BΔ*luxS* was significantly higher than the bacteria detected in both 60B (*P* < 0.0001) and 60CSF (**Figure 8**; *P* < 0.05). There was no significant difference between the number of 60CSFΔ*luxS* and WT 60CSF pneumococci present in the ears of mice, with the 60CSFΔ*luxS* strain proliferating in this niche at a slightly higher number compared to the wildtype.

Interestingly, in the lungs of mice infected with 60CSFΔ*luxS*, there was a significantly higher bacterial burden compared to mice challenged with 60CSF (**Figure 8**; *P* < 0.05). A similar trend was observed in the lungs of mice infected with 60BΔ*luxS*, although the difference in bacterial numbers recovered from this niche did not reach statistical significance.

## 4 Discussion

The LuxS/AI-2 QS system is dependent on the universal Autoinducer-2 (AI-2) signaling molecule, produced by LuxS. Previous studies on the pneumococcal LuxS QS system have shown that a *luxS* mutant strain is defective in biofilm formation and is less able to cause invasive diseases compared with wildtype pneumococci in the laboratory strain D39 (Trappetti et al., 2011b; Vidal et al., 2011). The opposite has been shown for the clinical ear isolate 947, where the *luxS* mutant was more able to cause invasive disease compared to the WT strain and no effect was observed on biofilm formation. In this work, we demonstrate that the different pathogenic profile previously observed in strains collected from a single pediatric patient (Agnew et al., 2022) might by linked to *luxS* and the restriction-modification (RM) locus *spnIII*.

*S. pneumoniae* is able to randomly switch its genomic DNA methylation pattern between six distinct states (*spnIIIA-F*) via recombination at the *spnIII* locus. Importantly, we previously found that these pneumococcal subpopulations exhibit phenotypic changes which have a major impact on bacterial virulence (Manso et al., 2014). This system, referred to as phasevarion (for phase-variable regulon), allows bacteria to generate a range of phenotypic variants within a population to act as an extra survival strategy (Phillips et al., 2019; Seib et al., 2020). Phasevarions have been characterized in multiple bacterial species such as *H. influenzae* (ModA) (Srikhanta et al., 2005; Fox et al., 2007; Atack et al., 2015), *M catarrhalis* (ModM) (Seib et al., 2002; Blakeway et al., 2014) the pathogenic *Neisseriae* (ModA, ModB, ModD) (Srikhanta et al., 2009; Seib et al., 2011; Seib et al., 2015; Tan et al., 2016), *Helicobacter pylori* (ModH) (Srikhanta et al., 2011), *Streptococcus suis* (Atack et al., 2018), and *Actinobacillus pleuropneumoniae* (Nahar et al., 2023). Analyzing the impact of altered methylase specificity on global transcriptomic patterns is complicated by the presence of multiple *spnIII* alleles in any culture. However, in a previous study, analysis of global gene expression was examined in a set of D39 *spnIII* mutants that were locked in a single specificity (Manso et al., 2014). RNA-seq identified only two major differences between the strains; the capsular polysaccharide serotype 2 biosynthesis operon *csp2* and the *luxS* gene, with both of these genes significantly downregulated in the *SpnIIIB-*locked mutant compared to all the other locked strains analyzed. Previously, we have shown that two clinical isolates 60B and 60CSF (serotype 15C, sequence type 8711) that were isolated from the blood and cerebral spinal fluid (CSF), respectively, of a single pediatric meningitis patient exhibit distinct virulence phenotypes in an intranasal murine infection model (Agnew et al., 2022). The two strains expressed similar amounts of capsular polysaccharide, but different ability to colonize the nasopharynx and the ear of mice. The first step in development of pneumococcal disease is the colonization of the human nasopharynx. *S. pneumoniae* is part of the commensal flora of the upper respiratory tract and shares this niche with other potentially pathogenic bacteria including Gram-negative bacteria such as *Haemophilus influenzae*, *Neisseria meningitidis*, and other Gram-positive bacteria, such as *Staphylococcus aureus*, and other streptococci (Faden et al., 1990). The nasopharyngeal flora is established in the first months of life (Faden et al., 1997) and it has a large turnover of colonizing species and serotypes that occupy the niche during the early years, but a balance is usually reached. In this community bacteria need to communicate to each other and thus Quorum sensing systems play a key role in this environment. In particular, the LuxS/AI-2 QS is the only QS shared between Gram-positive and Gram-negative bacteria. Apart from its role in quorum sensing, the enzyme LuxS is tightly coupled to the S-adenosylmethionine (SAM) utilization pathway. The metabolic enzyme LuxS synthesizes AI-2 as a by-product of the conversion of *S*-ribosyl-homocysteine to homocysteine, an integral reaction of the activated methyl cycle (AMC). Thus, the LuxS enzyme has dual roles, firstly as a critical enzyme in the AMC and secondly as an enzyme that produces the QS molecule AI-2. Homocysteine can be generated from S-adenosyl homocysteine (SAH) through two independent pathways: A) Most Gram-positive and Gram-negative bacteria use the enzyme Pfs to first produce *S*-ribosylhomocysteine (SRH), then LuxS catalyzes the conversion to homocysteine, simultaneously generating 4,5-dihydroxy-2,3-pentanedione (DPD), which is spontaneously converted to AI-2. B) Alternatively, bacteria such as *P. aeruginosa* use the enzyme SAH hydrolase (SahH) to generate homocysteine directly without generating DPD. Despite intensive research on luxS and AI-2 in the past few years, a clear separation of the possible metabolic role of AI-2 from its signaling activity, or by AMC metabolic pathway disruption, could not be achieved. In our previous paper we have shown chemical complementation of a D39Δ*luxS* mutant with exogenous AI-2 and identified the AI-2 receptor (Trappetti et al., 2017). Thus, unequivocally proving the role of AI-2 as a QS system in *S. pneumoniae* D39 strain.

In this current study, we investigated the role of *luxS* in the clinical isolates, 60B and 60CSF (Agnew et al., 2022). We quantified the *spnIII* alleles of the bacteria recovered from the nasopharynx of mice intranasally challenged with either 60B or 60CSF strains. Interestingly, bacteria recovered from mice infected with 60B had a higher proportion of the *spnIIIB* allele, corresponding to a low expression level of *luxS*, previously observed in the SpnD39IIIB locked strain (Manso et al., 2014). In contrast bacteria isolated from mice infected with 60CSF, had higher proportions of the *spnIIIA* and *spnIIIB* alleles. Importantly, when the *luxS* gene was deleted from 60B and 60CSF, the mutants had significantly reduced proportions of *spnIIIA* and *spnIIIB* and now had predominantly *spnIIIC.* These results indicate that the presence of LuxS is essential for the *spnIII* locus to switch to the *spnIIIA* and *spnIIIB* alleles, with the complete absence resulting in a switch to *spnIIIC* and *spnIIID*. The exact nature of this link between LuxS and the *spnIII* locus will require further characterization, to establish the mechanism by which LuxS influences the switching of alleles. In addition to differences in *spnIII* allele proportions, 60BΔluxS showed reduced ability to grow in media with only galactose. The opposite was true for 60CSF where deletion of the *luxS* gene improved the growth. Therefore, distinct mechanisms seem to be operating in these strains. In the case of 60B, mutation in the *luxS* gene decreases its ability to grow (as previously observed for the virulent strain D39 and for the ear isolate 947), while in 60CSF, deletion of *luxS* increased the ability of the strain to grow in galactose media. This suggested a strain-specific effect of *luxS* in these clinical isolates. When the ability of the strains to form a biofilm was tested, we found that both strains could form biofilm at comparable rates, but again *luxS* mutation was deleterious for the 60B strain, whilst improving the biofilm formation capacity of the 60CSF strain. Addition of AI-2 improved the biofilm capacity in both WT strains, while interestingly chemical complementation of the *luxS* mutants with exogenous AI-2 could not be achieved. Thus, LuxS seems to have more of a metabolic role rather than a role in QS in these clinical isolates, dissimilar to the findings in D39 (Trappetti et al., 2017). In a murine infection model the 60BΔ*luxS* strain displayed an enhanced transit to the ear compared to the WT, and increased transit to the lungs for the 60CSFΔ*luxS* strain (**Figure 8**). An increased transit of the 60BΔ*luxS* strain to the ear together with the reduced capacity of the strain to form a biofilm, suggests that functional *luxS* may act to facilitate adherence to the nasopharynx, but may interfere with transit to other host niches, like the ear compartment. However, in the 60CSF strain, deletion of *luxS* increased its ability to form a biofilm and also increased the ability of the strain to persist in the lung.

These results suggested an opposite role for *luxS* in the infection profiles of 60B in comparison to 60CSF, with a potential role in metabolism. We then analyzed the metabolic profiles of the *luxS* mutants and importantly, both *luxS* mutants displayed the same metabolic profile, having lost the ability to metabolise psicose and having acquired the ability to metabolise fucose in comparison to the wildtype. Most importantly, strains recovered from the nasopharynx of mice intranasally infected with 60B or 60CSF displayed the same changes in metabolic profile as the *luxS* mutants, indicating that *luxS* may be downregulated in these strains during nasopharyngeal colonization. Our earlier analysis of the *spnIII* allele variants in 60B and 60CSF isolated from murine nasal tissue indicated both had high proportions of *spnIIIB*, which is associated with increased colonization ability and downregulation of *luxS.* Therefore, during colonization of the nasopharynx 60B and 60CSF undergo changes to increase their proportion of *spnIIIB* allele to aid their colonization, likely resulting in a downregulation of *luxS*, thereby altering their ability to metabolise fucose and psicose in a similar way as was observed for the *luxS* mutants. Furthermore, other factors may be contributing to the observed different phenotypes that have yet to be identified. It is important that previous and future studies on pneumococcal *luxS* and its involvement in virulence be interpreted in the context of the potential switching between epigenetically-different subpopulations during the course of any experimental infection study.

## Data availability statement

The raw data supporting the conclusions of this article will be made available by the authors, without undue reservation.

## Ethics statement

The animal study was reviewed and approved by University of Adelaide Animal Ethics Committee.

## Author contributions

Conceptualization, HA, EB, JP, and CT; methodology, HA, JA, AF, SW, and CT; investigation, HA, JA, AF, MvdL, and CT; synthesis of AI-2/DPD, ES and AA; data curation, HA and CT; writing—original draft preparation, HA, EB, JP, and CT. All authors contributed to the article and approved the submitted version.
