# Supplementary material for: Uncovering the link between the SpnIII restriction modification system and LuxS in Streptococcus pneumoniae meningitis isolates
Source: Front Cell Infect Microbiol. 2023 May 1;13:1177857. doi: 10.3389/fcimb.2023.1177857 (PMC10184825; doi:10.3389/fcimb.2023.1177857)
Supplement: Supplementary file 1 [file DataSheet_1.docx]

Supplementary Material

Uncovering the link between the SpnIII restriction modification system and LuxS in *Streptococcus pneumoniae* meningitis isolates.

Hannah N Agnew^1^, John M Atack^2 3^, Ann RD Fernando^1^, Sophie N Waters^1^, Mark van der Linden^3^, Erin Smith^5^, Andrew D. Abell^5^, Erin B Brazel^1^, James C Paton^1*^, Claudia Trappetti^1*^

*** Correspondence:** claudia.trappetti@adelaide.edu.au, james.paton@adelaide.edu.au

# Supplementary Data

**Synthesis of AI-2 (DPD)**

Starting materials were obtained from commercial sources and used without further purification unless otherwise stated. Thin layer chromatography (TLC) was performed on Merck aluminium sheets with silica gel 60 F_254_ silica plates. The compounds were visualised with an Oliphant (6W 254 nm tube) UV lamp, vanillin stain and/or potassium permanganate stain. Flash chromatography was carried out using Merck Kiselgel 60 (230-400 mesh). All yields are reported as isolated yields as judged to homogenous by TLC and NMR spectroscopy. NMR spectra were recorded using either an Agilent DD2 console 500 MHz or a Varian Inova 600 MHz spectrometer. NMR spectra were reference to their respective solvents: chloroform-*d* (CDCl_3_, ^1^H δ 7.26 ppm, ^13^C δ 49.3 ppm); water-*d*_2_ (D_2_O, ^1^H 4.65 ppm)

Dimethyl amine (10 mL, 33vol% in ethanol) was added to (-)-methyl (S)-2,2-dimethyl-1,3-dioxalane-4-carboxylate (0.49 mL, 3.38 mmol) at 0°C and stirred for 3 hours before being placed in the fridge (5 $^{\circ}$C) for 3 days. The volatile compounds were evaporated, and the residue was subjected to flash chromatography. Elution with 1:1 EtOAC in hexanes produced *N*,*N*-dimethyl (S)-α,β-isopropylideneglyceramide as an oil (0.449 g, 77%).

^1^H NMR (500 MHz, CDCl_3_) δ 4.69 (t, *J* = 6.6 Hz, 1H), 4.38 (dd, *J* = 8.4, 6.5 Hz, 1H), 4.14 (dd, *J* = 8.5, 6.8 Hz, 1H), 3.12 (s, 3H), 2.97 (s, 3H), 1.42 (d, *J* = 1.4 Hz, 6H) ppm.

*N*,*N*-dimethyl (S)-α,β-isopropylideneglyceramide (0.440 g, 2.54 mmol) was dissolved in anhydrous ether (10 mL) under nitrogen and cooled to 0$^{\circ}$C. Isopropenylmagnesium bromide (5.33 mL, 2.67 mmol, 0.5M in THF) was added slowly. The resulting mixture was stirred for 10 min before sulfuric acid solution (1M) was added dropwise until the precipitate redissolved. The aqueous layer was extracted with ether (x3) and the combined organic extracts were neutralised over solid sodium carbonate, dried over anhydrous magnesium sulfate, filtered and concentrated under reduced pressure. The residue was subjected to flash chromatography. Elution with 20% EtOAC in hexanes afforded (S)-4-methacryloyl-2,2-dimethyl-1,3-dioxolane as an oil (0.166 g, 38%).

^1^H NMR (500 MHz, CDCl_3_) δ 6.04 (d, *J* = 1.2 Hz, 1H), 5.92 (d, *J* = 1.5 Hz, 1H), 5.07 (dd, *J* = 7.3, 6.1 Hz, 1H), 4.23 (dd, *J* = 8.5, 7.3 Hz, 1H), 4.10 (dd, *J* = 6.4, 5.9 Hz, 1H), 1.91 (s, 1H), 1.44 (s, 2H), 1.43 (s, 3H).

(S)-4-methacryloyl-2,2-dimethyl-1,3-dioxolane (0.05 g, 0.24 mmol) was dissolved in THF (1mL) and water (1 mL) and cooled to 0°C. TFA (1 mL) was added dropwise and the resulting mixture was stirred for 1.5 hrs. The volatiles were concentrated under reduced pressure. The residue was subjected to flash chromatography. Elution with 5% MeOH in DCM afforded (S)-1,2-dihydroxy-4-methyl-4-penten-3-one as a colourless liquid (0.015 g, 41%).

1H NMR (500 MHz, CDCl_3_) δ 5.96 (d, J = 1.5 Hz, 1H), 5.95 (s, 1H), 4.91 (m, 1H), 3.95 (m, 1H), 3.71 (dd, J = 11.6, 4.8 Hz, 1H), 3.01 (s, 2H), 1.95 (s, 3H).

(S)-1,2-dihydroxy-4-methyl-4-penten-3-one (15 mg, 0.12 mmol) was dissolved in MeOH (10 mL) and cooled in an acetone-dry ice bath (-78 $^{\circ}$C). Ozone was bubbled through the solution until it turned blue (approx. 2 min). Oxygen gas was then bubbled through until the ozone was removed and dimethyl sulphide was added. The mixture was allowed to come to room temperature and stirred overnight.

The bulk of the material was added water (3 mL) and concentrated to remove the volatiles. Diluted with 1M potassium phosphate buffer to give pH 7.3 and final concentration 5 mM product in 0.5M potassium phosphate buffer.

**Supplementary Table 1: Phenotype microarray results for 60B, 60CSF, *luxS* mutants and isolates recovered from murine noses 24 h post-infection in Biolog PM1 plate.** Catabolism was measured through a colorless tetrazolium dye being reduced by NADH produced during catabolism. +, metabolism occurred; -, no metabolism occurred. Metabolism was determined by calculating the change in OD_590_ from the initial (0 h) to final (24 h) measurements. These values were then compared with the change in the negative value and an arbitrary value based on the change in the negative control was used to determine if catabolism occurred. Carbon sources in which there were no differences in metabolism between the WT, mutant or strains recovered from mice are not shown.

| **Carbon source** | **60B** | **60CSF** | **60B∆luxS** | **60CSF∆luxS** | **60B (mouse)** | **60CSF (mouse)** |
| --- | --- | --- | --- | --- | --- | --- |
| **L-Fucose** | - | - | + | + | + | + |
| **D-Psicose** | + | + | - | - | - | - |


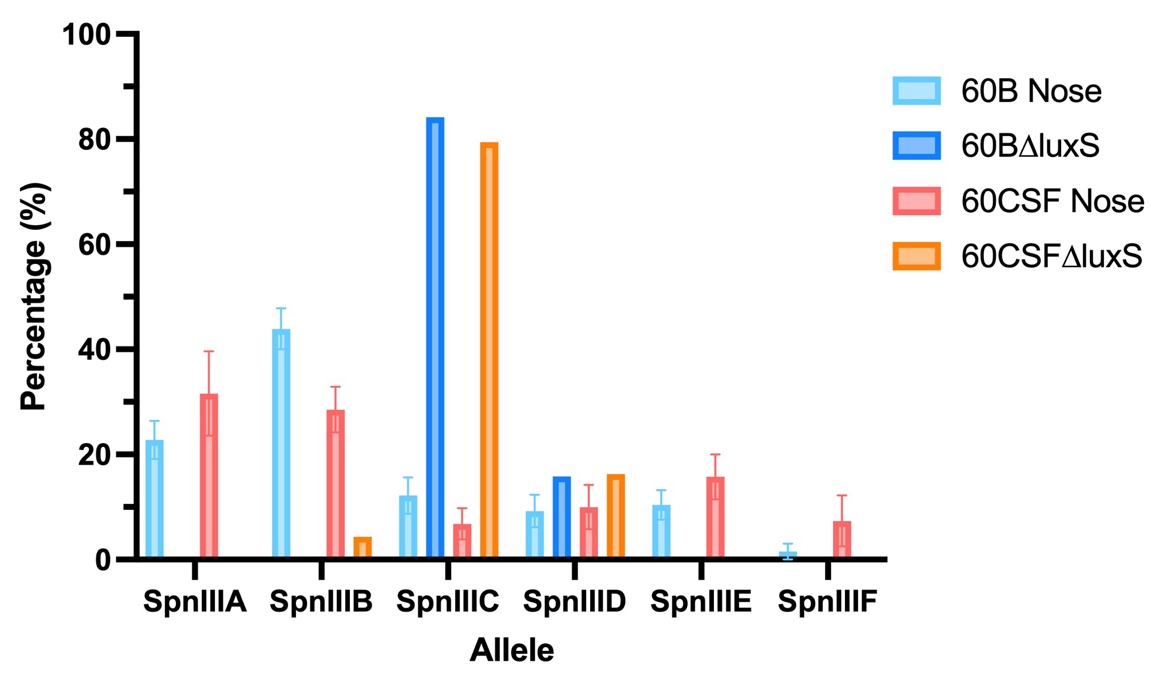


**Supplementary Figure 1: *spnIII* allele frequencies of *luxS* mutants, and 60B and 60CSF recovered post intranasal murine infection.** Mice were intranasally challenged with 10^8^ CFU *S. pneumoniae* serotype 15C ST8711 blood isolate (60B) or CSF isolate (60CSF). At 24 h, mice from each group were humanely euthanized and pneumococci in the nasal tissue were harvested. *spnIII* allele quantification was performed on DNA extracted from colonies grown from nasopharynx samples, in addition to 60B∆luxS and 60CSF∆luxS colonies grown on BA plates overnight at 37°C with 5% CO_2_. Allele percentages are displayed for WT strains recovered from the nose of mice (Figure 1) and *luxS* mutants from frozen stock.


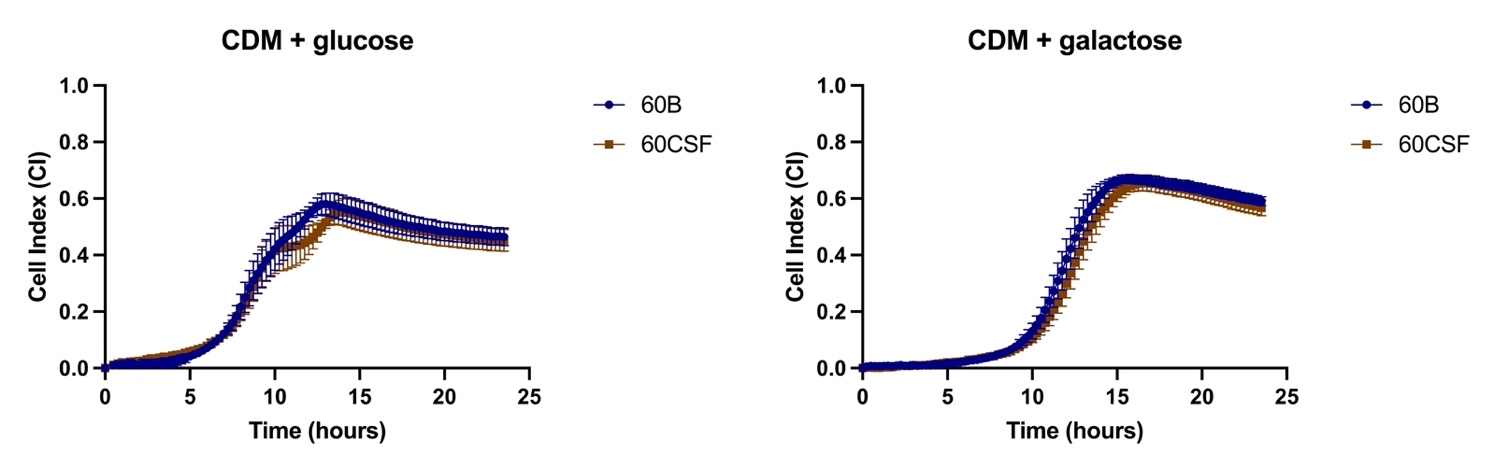
**
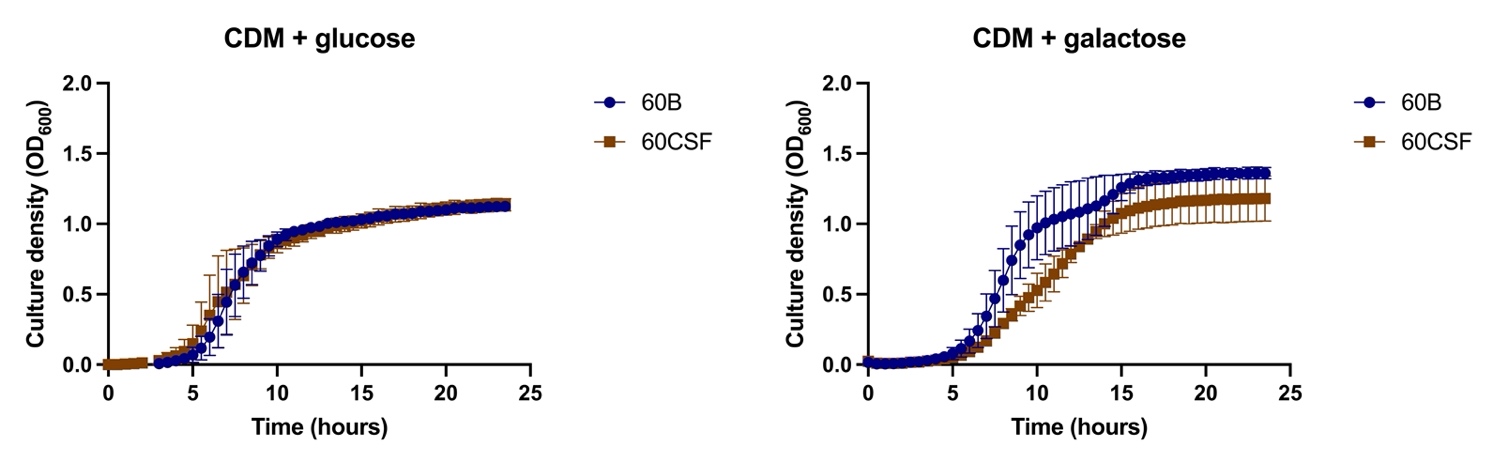
Supplementary Figure 2: Growth of 60B and 60CSF in CDM + glucose and CDM + galactose.** Isolates were grown in 200 µL CDM supplemented with 0.5% glucose (CDM + glucose) or 0.5% galactose (CDM + galactose). OD_600_ was measured every 30 min for 24 h. Data are mean OD_600_ ± standard error mean (SEM) from two independent assays, each performed in triplicate. Significance of differences in final OD and mid-exponential phase OD between strains was determined using two-tailed Student’s *t* test.

**Supplementary Figure 3: Biofilm formation of 60B and 60CSF in CDM + glucose and CDM + galactose.** Bacteria were cultured overnight on BA plates before being diluted to a final OD_600_ of 0.05 in CDM + 0.5% glucose or CDM + 0.5% galactose. 200 µL of each bacterial strain in each culture medium was placed into wells of a xCELLigence E-plate, with the plate being placed in the cradles of the RTCA-DP platform and incubated at 37°C with 5% CO_2_. Biofilm formation was determined by measuring cell index (CI) every 15 min over 24 h using real-time cell analysis (RTCA) xCELLigence technology. Data are mean CI ± standard error mean (SEM) from three independent assays, each performed in triplicate.


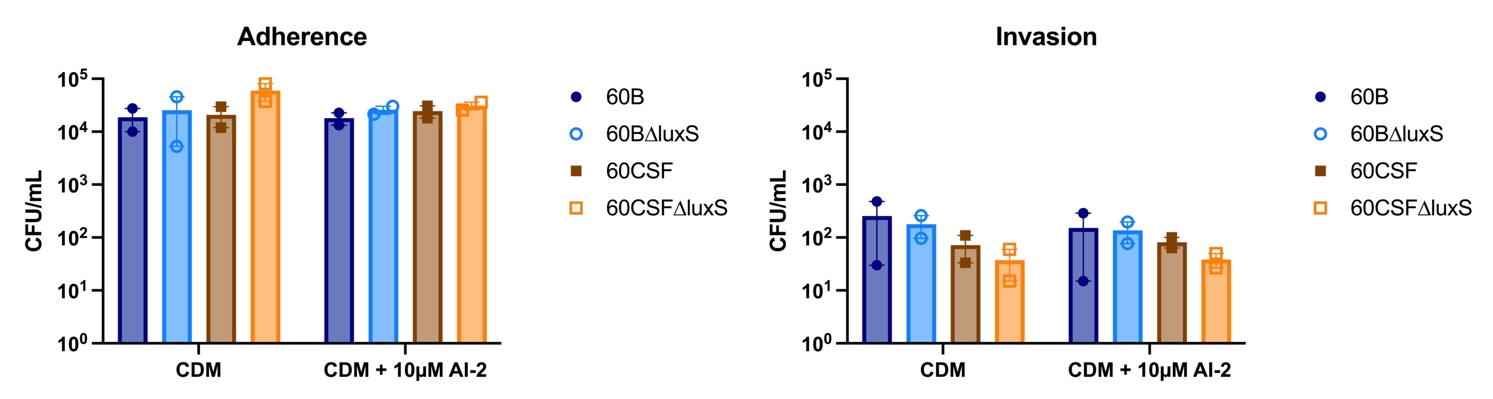


**Supplementary Figure 4: Adherence and invasion of 60B, 60CSF, 60B∆luxS and 60CSF∆luxS strains to Detroit 562 cells.** Strains at OD_600_ 0.2 were inoculated onto monolayers of Detroit 562 cells in CDM ± 10µM AI-2, and incubated for 2 h before being tested for adherent and invasive bacteria (see Materials and Methods). Data are mean adherent or invasive bacteria ± standard error mean (SEM) from two independent assays, each performed in triplicate. Statistical analysis was performed using two-tailed Student’s *t* test. No significant differences were detected between the adherence and invasion of the strains in different conditions.


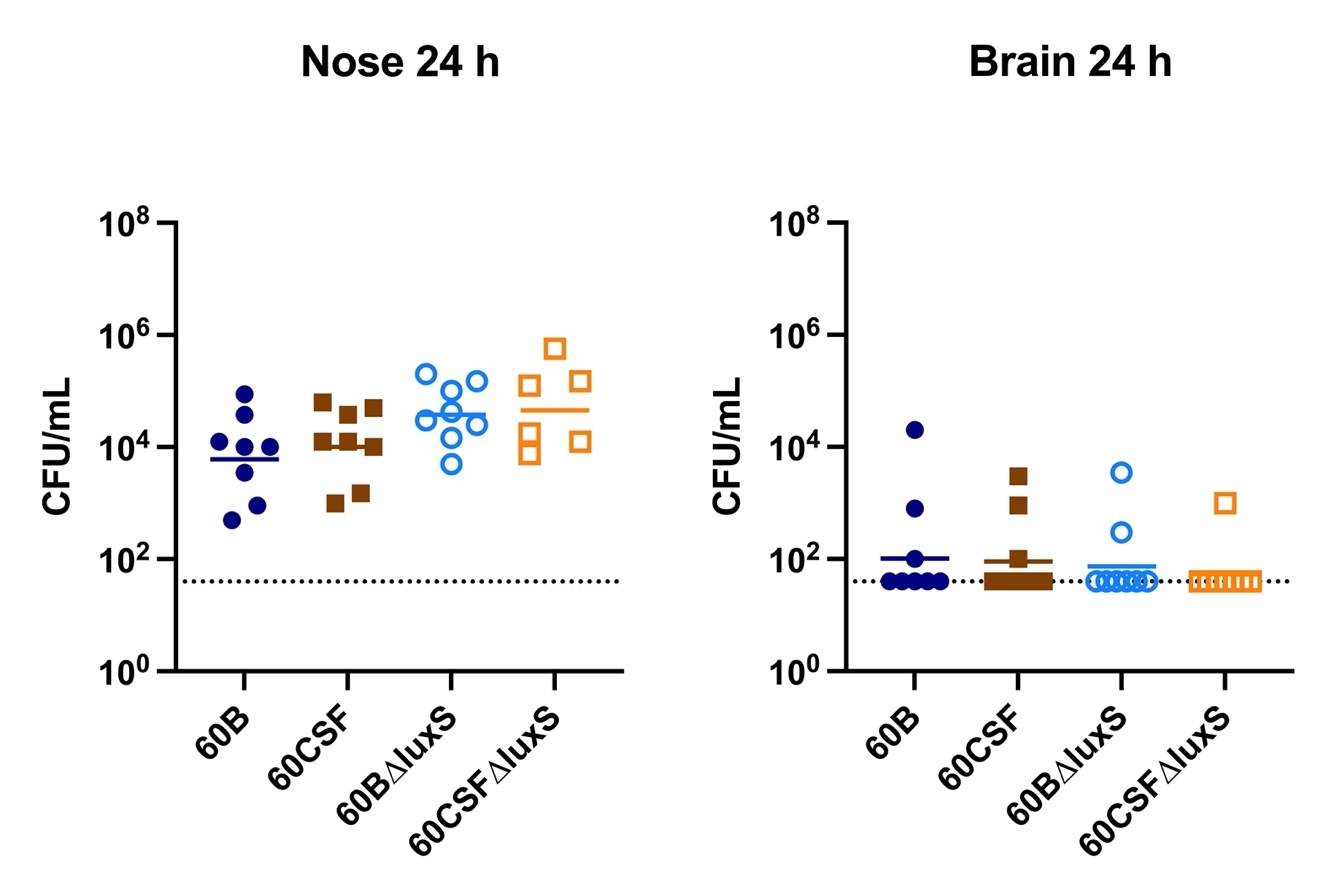


**Supplementary Figure 5: Murine brain and nose bacterial burden post-infection with 60B, 60CSF, 60B∆luxS and 60CSF∆luxS strains.** Groups of 8 mice were infected intranasally with 10^8^ CFU of the indicated strain. At 24 h, mice from each group were humanely euthanized and pneumococci in the brain, ear, lungs and nasal tissue were enumerated. Viable bacterial counts are displayed for each mouse in the brain and nose (ears and lungs are in manuscript); horizontal bars indicate the geometric mean (GM) CFU for each group; the dotted line indicates the detection threshold. No significant differences in bacterial load between groups were detected.
